# A Two Fiber Bragg Gratings Sensing System to Monitor the Torque of Rotating Shaft

**DOI:** 10.3390/s16010138

**Published:** 2016-01-21

**Authors:** Yongjiao Wang, Lei Liang, Yinquan Yuan, Gang Xu, Fang Liu

**Affiliations:** 1 National Engineering Laboratory for Fiber Optic Sensing Technology, Wuhan University of Technology, Wuhan 430070, China; wangyongjiao@126.com (Y.W.); l30l30@126.com (L.L.); xugang524@163.com (G.X.); fangliu@whut.edu.cn (F.L.); 2 School of Computer Science and Engineering, Henan University of Urban Construction, Pingdingshan 467000, China

**Keywords:** fiber Bragg grating, rotating shaft, torque and torsion angle, vibration monitoring

## Abstract

By fixing two FBGs on the surface of a rotating shaft along the direction of ±45° and using dynamic wavelength demodulation technology, we propose an optical fiber sensing system to monitor the driving torque and torsion angle of the rotating shaft. In theory, the dependence relation of the dynamic difference of central wavelengths on the torque and torsion angle of the rotating shaft has been deduced. To verify an optical fiber sensing system, a series of sensing experiments have been completed and the measured data are approximately consistent with the theoretical analysis. The difference of two central wavelengths can be expressed as the sum of two parts: a “DC” part and a harmonic “AC” part. The driving torque or torsion angle is linear with the “DC” part of the difference of two central wavelengths, the harmonic “AC” part, meaning the torsion angle vibration, illustrates that periodic vibration torque may be caused by inhomogeneous centrifugal forces or inhomogeneous additional torques produced by the driving system and the load.

## 1. Introduction

Rotating machinery, such as electromotors, generators, steam turbines, compressors and aviation engines, plays an important role in modern industrial development, and is also key equipment in military and civilian fields. The health status and faults of rotating machinery can be understood by monitoring the vibration signals of the rotating shaft of the rotating machinery, and the vibration signals of the rotating shaft are important indicators for equipment safety assessments [1]. In order to ensure the healthy running of rotating machinery, it is necessary to develop an efficient monitoring method. Electrical sensors, such as eddy-current sensors and inductive sensors are often used for the vibration measurement of rotating shaft, but electrical sensors are susceptible to electromagnetic interference [2].

Compared with electrical sensors, fiber Bragg grating (FBG) sensors have unique advantages such as immunity to electromagnetic fields, compatibility with harsh environmental conditions, multiplexing capabilities, high sensitivity, small volume, light weight and low noise. Therefore, FBGs are becoming widely used in health monitoring, vibration measurement, and other areas and are attracting more and more attention in diverse application [3,4,5,6]. Antunes proposed an L-shaped beam-based FBG sensor to monitor structure vibration [7], Weng constructed a diaphragm-based acceleration sensor for structure health monitoring [8], Liu presented a flat diaphragm-based FBG sensor to determine the vibration acceleration of a measured body [9], Wang proposed a non-contact magnetic coupling FBG sensor to achieve non-contact measurements of object displacement [10] and Tan presented a non-contact vibration FBG sensor to monitor the vibration of rotating shafts [11]. Hwang and Lee proposed an online torsion sensing method using FBG sensors and an optical coupling method to monitor the torque of a rotating shaft [12,13], but the optical power measurement approach is susceptible to the interference of light loss caused by the relative movement of the optical coupler, moreover the quantitative relation between the optical power and torque was not shown. In this paper, based on the wavelength measurements of two FBGsby a high speed demodulator [14], we propose a system for monitoring the torque and torsion angle of rotating shafts, and we have completed a serial of vibration experiments, demonstrating that the measured data are identical with the simulated results.

## 2. Theory for the Wavelength Measurement of Two FBGs

**Figure 1:**
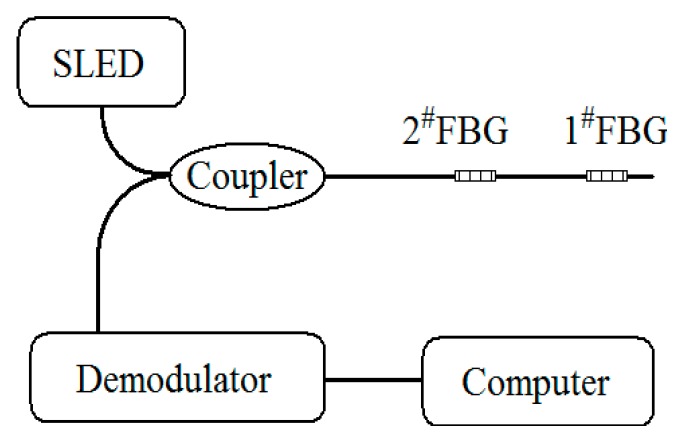
Experimental scheme.

**Figure 2:**
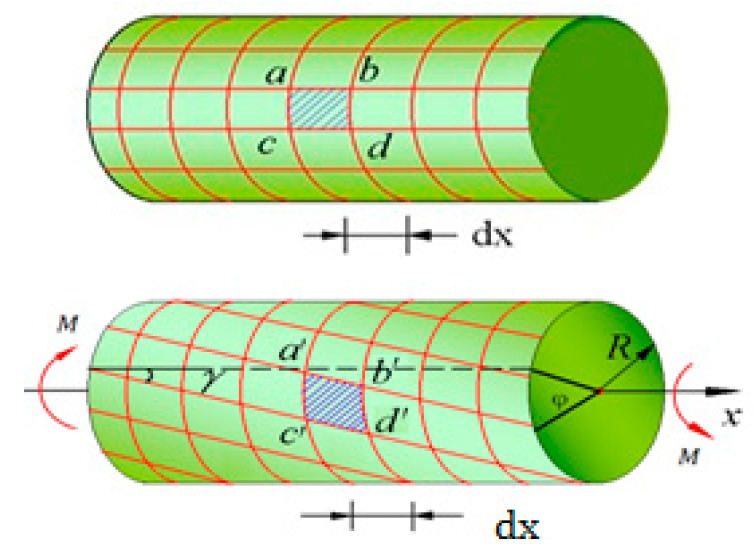
Sketch of the shaft torsion.

The experimental scheme is illustrated in Figure 1. The system consists of a super light-emitting diode (SLED) (central wavelength ≈1550 nm, spectrum width ≈60 nm), a 3dB coupler, two FBGs (1^#^FBG and 2^#^FBG), a dynamic demodulator and a computer. The two FBGs are pasted along the 45° direction on the surface of the transmission shaft. Through the coupler, the light emitted from the SLED arrives at the gratings, and the reflected light from the two gratings is directed to the dynamic demodulator. Figure 2 is a sketch of the transmission shaft, whose diameter and length are denoted by *D* and *L* respectively. Under the action of pure torque *M*, the maximum shear strain occurs on the surface of the circular shaft and in the direction of 45° related to the axial direction:

(1) $$\varepsilon =\frac{D}{2EI_{p}}M$$

where *EI*_p_ is torsional stiffness. Correspondingly, the torsion angle of the shaft with length *L* is:

(2) $$\varphi =\frac{L}{GI_{p}}M$$

For a solid cylinder, using tensile elastic modulus *G* and Poisson’s ratio μ, the shear elastic modulus *G* and inertia moment *I*_p_ can be expressed as:

(3) $$G=\frac{E}{2(1+\mu )}$$

(4) $$I_{P}=\frac{\pi D^{4}}{32}$$

**Figure 3:**
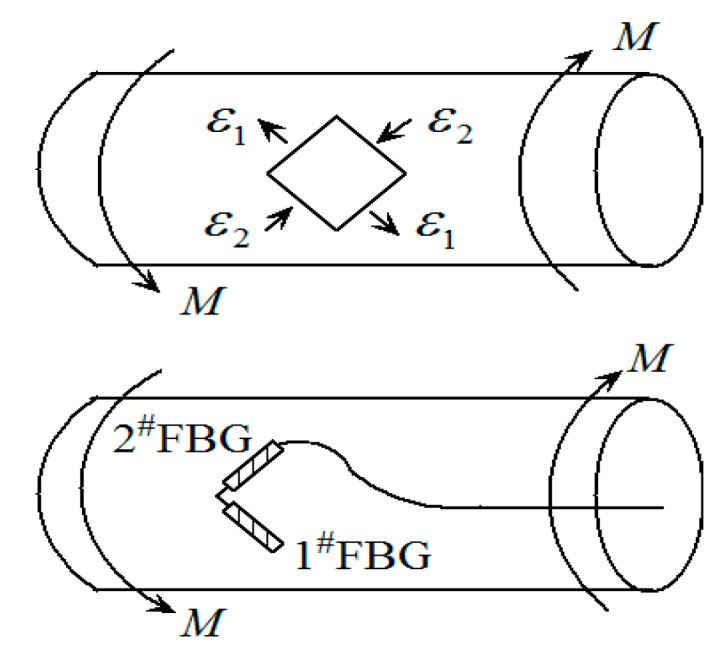
Two FBGs pasted along the maximum strain of the shaft torsion.

The reflection spectra of two FBGs undergoing dynamic uniform strain are symmetrical about their central wavelengths, and the dependence relationship of the wavelength on the strain and temperature change can be written as [3,4,5,6]:

(5) $$\lambda_{1}(t)=\lambda_{10}+\lambda_{10}(1-p_{e})\varepsilon_{1}(z_{\mathrm{mid}},t)+\lambda_{10}[\zeta_{s}+(1-p_{e})(\alpha_{s}-\alpha_{f})]\Delta T$$

(6) $$\lambda_{2}(t)=\lambda_{20}+\lambda_{20}(1-p_{e})\varepsilon_{2}(z_{\mathrm{mid}},t)+\lambda_{20}[\zeta_{s}+(1-p_{e})(\alpha_{s}-\alpha_{f})]\Delta T$$

where $\varepsilon_{1}(z_{\mathrm{mid}},t)$ and $\varepsilon_{2}(z_{\mathrm{mid}},t)$ are respectively the strains at the midpoints of two FBGs pasted along the direction of 45° on the surface of circular shaft as shown in Figure 3, so $\varepsilon_{1}(z_{\mathrm{mid}},t)\;=-\varepsilon$, $\varepsilon_{2}(z_{\mathrm{mid}},t)\;=\varepsilon$. Δ*T* is the temperature change, λ_10_ and λ_20_ are the initial wavelengths of two FBGs without the strain, *p*_e_ = 0.22 is the effective elastooptic parameter of optical fiber. ζ_s_ = 6.45 × 10^−6^/°C is the thermo-optic coefficient of fiber core, α_s_ = 11.6 × 10^−6^/°C and α_f_ = 0.55 × 10^−6^/°C are the linear expansion coefficients of fiber core and rotating shaft respectively. Taking *λ*_10_ = 1540.619 nm and *λ*_20_ = 1555.791 nm, we obtained $\lambda_{10}[\zeta_{s}+(1-p_{e})(\alpha_{s}-\alpha_{f})]=23.37$ pm/°C and $\lambda_{20}[\zeta_{s}+(1-p_{e})(\alpha_{s}-\alpha_{f})]=23.60$ pm/°C, so the effect of temperature change on the central wavelengths of two FBGs cannot be omitted for small strain, but the effect of temperature change on their difference is very little and can be omitted. Then the dynamic difference of central wavelengths of two FBGs undergoing dynamic linear strain is:

(7) $$\Delta \lambda_{21}(t)=(\lambda_{20}-\lambda_{10})+\lambda_{20}(1-p_{e})\varepsilon_{2}(z_{\mathrm{mid}},t)-\lambda_{10}(1-p_{e})\varepsilon_{1}(z_{\mathrm{mid}},t)\;$$

(8) $$=(\lambda_{20}-\lambda_{10})+(\lambda_{10}+\lambda_{20})(1-p_{e})\varepsilon$$

Considering Equations (1)–(4), we have:

(9) $$\Delta \lambda_{21}(t)=(\lambda_{20}-\lambda_{10})+(\lambda_{10}+\lambda_{20})\left(1-P_{e}\right)\frac{D}{2EI_{p}}M(t)$$

and:

(10) $$\Delta \lambda_{21}(t)=(\lambda_{20}-\lambda_{10})+(\lambda_{10}+\lambda_{20})\left(1-P_{e}\right)\frac{D}{4(1+\mu )}\frac{\varphi (t)}{L}$$

Equations (9) and (10) show that the wavelength difference is linear with the torque and torsion angle. Since our experiments show that the wavelength difference vibrates periodically with time, so we can suppose that the torsion angle of shaft obeys the simple harmonic vibration too:

(11) $$\varphi (t)/L=\varphi_{\mathrm{DC}}+\varphi_{m}\sin(2\pi ft+\phi_{0})$$

(12) $$M(t)=M_{\mathrm{DC}}+M_{m}\sin(2\pi ft+\phi_{0})$$

where $\varphi_{\mathrm{DC}}$ is the “DC” part of the torsion angle, $\varphi_{m}$ is the maximum of the “AC” part of the torsion angle, *M*_DC_ is the “DC” part of the torque, *M*_m_ is the maximum of the “AC” torque, and they have the following relationship:

(13) $$M_{\mathrm{DC}}=GI_{p}\varphi_{\mathrm{DC}}\;,\;M_{m}=GI_{p}\varphi_{\mathrm{DC}}\;$$

Defining two factors:

(14) $$K_{\varphi }=(\lambda_{10}+\lambda_{20})\left(1-P_{e}\right)\frac{D}{4(1+\mu )}$$

(15) $$K_{M}=(\lambda_{10}+\lambda_{20})\left(1-P_{e}\right)\frac{D}{2EI_{p}}$$

Then we have the following relationships about the dynamic difference of central wavelengths of two FBGs:

(16) $$\Delta \lambda_{21}(t)=\Delta \lambda_{\mathrm{DC}}\;+\Delta \lambda_{m}\sin(2\pi ft+\phi_{0})$$

where the “DC” part and the maximum of “AC” part are respectively:

(17) $$\Delta \lambda_{\mathrm{DC}}\;=(\lambda_{20}-\lambda_{10})+K_{\varphi }\varphi_{\mathrm{DC}},\;\Delta \lambda_{m}=K_{\varphi }\varphi_{m}$$

or be expressed by the torque:

(18) $$\Delta \lambda_{\mathrm{DC}}\;=(\lambda_{20}-\lambda_{10})+K_{M}M_{\mathrm{DC}},\;\Delta \lambda_{m}=K_{M}M_{m}$$

Progressively, the “DC” part of torque *M*_DC_ comes from two parts, one is the driving torque, and the other is the friction torque between the shaft and the bearings:

(19) $$M_{\mathrm{DC}}=M_{f}+M_{d}$$

thus the central wavelength difference of two FBGs undergoing driving torque can be denoted by:

(20) $$\Delta \lambda_{\mathrm{DC}}\;(M_{d})=\Delta \lambda_{\mathrm{DC}}(0)+k_{M}M_{d}$$

(21) $$\Delta \lambda_{\mathrm{DC}}(0)\;=(\lambda_{20}-\lambda_{10})+k_{M}M_{f}$$

## 3. Experimental Setup and Results

**Figure 4:**
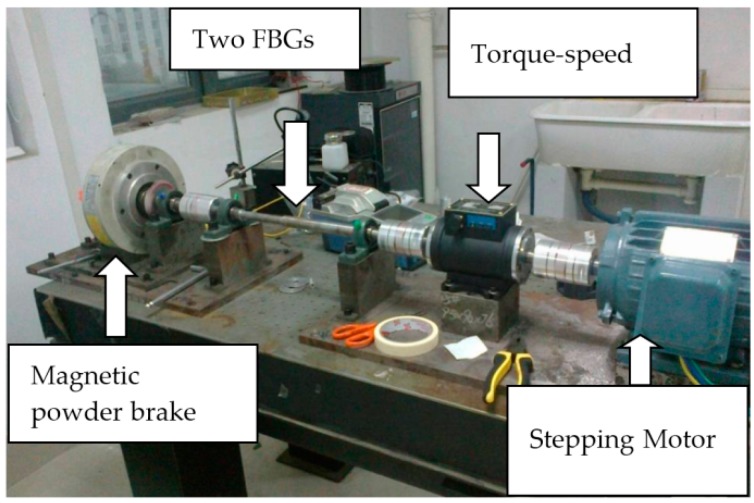
Experimental setup.

Figure 4 shows the basic experimental setup. The power is provided by a stepping motor, and the rotation speed of the shaft may be adjusted by the transducer. The load is provided by a magnetic powder brake and its torque can be changed from 0 to 3.4 Nm by adjusting the input current (0–0.45 A). Between the motor and the load we have installed a torque-speed sensor which can measure the torque and rotation speed. The optical system consists of two FBGs connected in series, a 3 dB coupler, and a SLED.A fiber optic rotary joint was used to transmit the light between the optical fiber at the shaft and the optical fiber on the ground. Through the coupler, the light emitted from the SLED arrives at the dual FBGs. For the wavelength demodulation scheme, the reflected light from the two gratings is directed to a high-speed demodulation instrument to monitor the dynamic Bragg wavelength of the two FBGs. The high-speed demodulation instrument (sm130 optical sensing interrogator, Micron Optics, Inc. Atlanta, GA, USA) can measure the peak wavelengths of two FBGs with a resolution of 1 pm and sampling frequency of 1 kHz.

**Figure 5:**
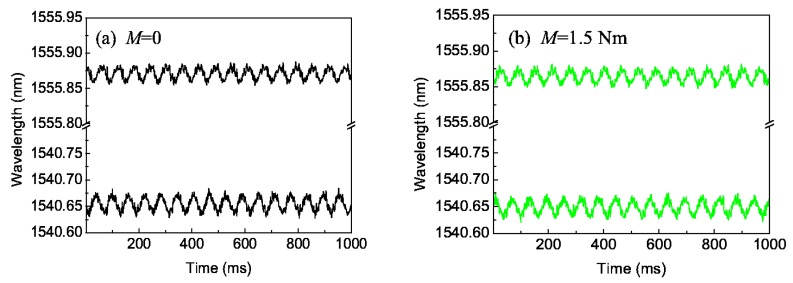
Central wavelengths of two FBGs with time: (**a**) *M* = 0; (**b**) *M* = 1.5 Nm.

We prepared two FBGs (denoted as 1^#^FBG and 2^#^FBG) with different initial wavelengths, and as shown in Figure 3, pasted them on the surface of the circular shaft and in the direction of ±45° relative to the axial direction, respectively. Without any strain, their initial wavelengths are λ_10_ = 1540.619 nm and λ_20_ = 1555.791 nm, the wavelength difference of 15 nm ensures the separation of the two reflection peaks in the monitoring process. First, we have completed a series of experiments to account for the effect of the driving torque, where the rotating speed of 960 r/min is fixed. Figure 5 gives the typical central wavelengths of two FBGs with time for two torques (0 and 1.5 Nm). It can be seen that the central wavelengths of 1^#^FBG and 2^#^FBG follows an approximate sine change with time shown by Equation (16), and their vibrating maximum Δλ_m_ are almost the same and the phase difference is about π; the deviations are because the two FBGs are not pasted strictly in the ±45° direction.

**Figure 6:**
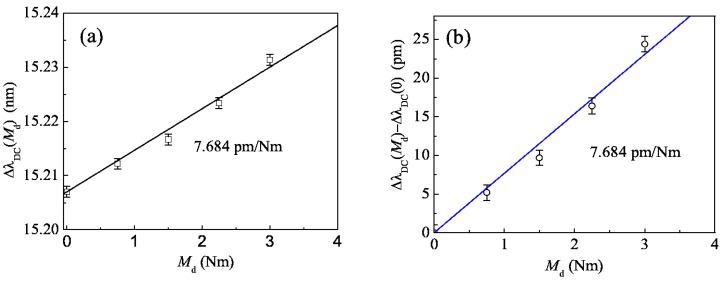
Central wavelength *versus* driving torque: (**a**) Δλ_DC_(*M*_d_); (**b**) Δλ_DC_(*M*_d_) − Δλ_DC_(0).

Figure 6a gives the values of Δλ_DC_ corresponding to different driving torques (0, 0.75, 1.5, 2.25 and 3.0 Nm) by squares, whereby one can see that these squares are nearly on one line. Substituting Equation (4) into Equation (15) and using the parameters of the shaft (45 steel, *E* = 210 GPa, *D* = 20 mm), we can obtain the theoretical value *k*_M_ = 7.684 pm/Nm in Equations (15), (18) and (21), and the line in Figure 6 was drawn according this slope. Noting that Δλ_DC_(0) = 15.207 nm and λ_20_ − λ_10_ = 15.172 nm, we can obtain that their difference Δλ_DC_(0) − (λ_20_ − λ_10_) ≈ 32 pm comes from the friction torque between the shaft and bearings, so we calculate the values of Δλ_DC_(*M*_d_) − Δλ_DC_(0) for the nonzero driving torque *M*_d_ as shown in Figure 6b, and it can be seen that the experimental data is approximately consistent with the theoretical analysis and Δλ_DC_(*M*_d_) − Δλ_DC_(0) is proportional to the driving torque. The deviation mainly comes from two reasons: One is because the two FBGs are not pasted strictly in the ±45°direction; the other is the error in driving torque caused by the current which controls the magnetic powder brake.

**Figure 7:**
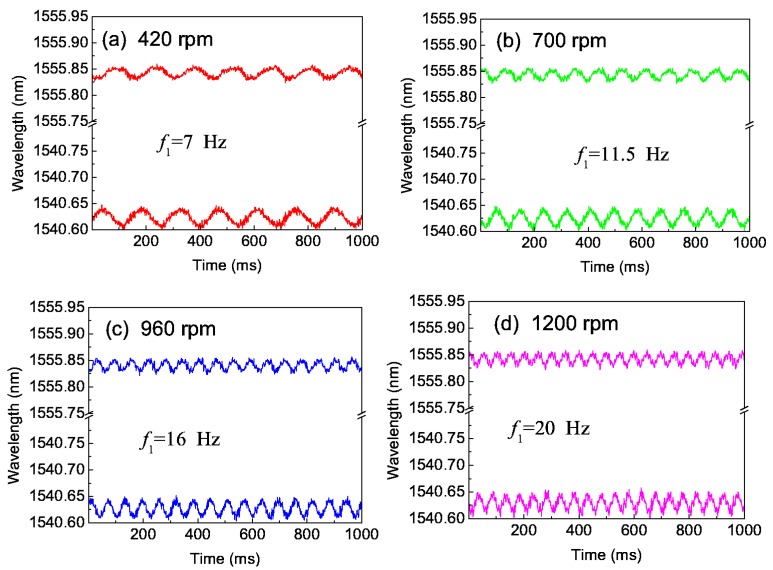
Central wavelengths of two FBGs with the time for four rotation speeds: (**a**) 420; (**b**) 700; (**c**) 960 and (**d**) 1200 rpm.

Then, we have investigated the effect of the rotation speed, where the torque is fixed. Figure 7a–c gives the typical central wavelengths of two FBGs with time for three rotation speeds (420, 700, 960 and 1200 rpm), where the lower wavelength-time curve is λ_1_(t) of 1^#^FBG, the upper wavelength-time curve is λ_2_(t) of 2^#^FBG,and *f*_1_ is the 1-order frequency of the fast Fourier transform for the vibration signal. We found that the values of Δλ_DC_ and Δλ_m_ are almost the same and the vibrating frequency of Δλ_1_(*t*) and Δλ_2_(*t*)increases with increasing rotation speed.

**Figure 8:**
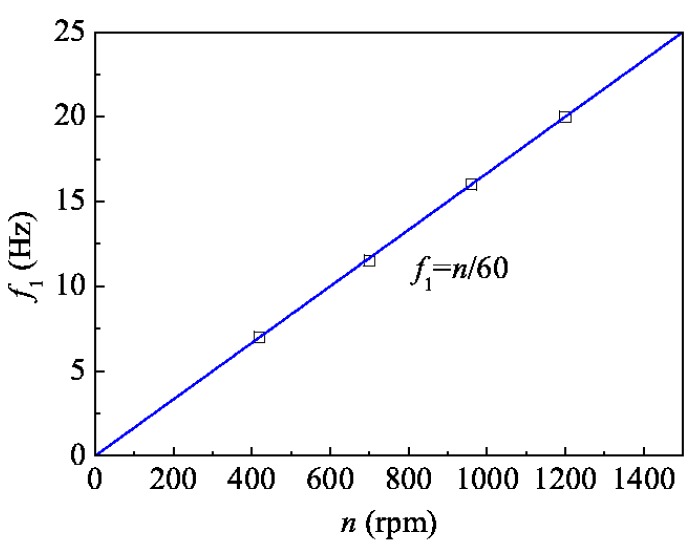
1-order frequency of vibration signal *versus* rotating speed.

Figure 8 gives the vibration frequency values corresponding to four different rotation speeds (420, 700, 960, 1200 rpm) by squares, the line was drawing according to the formula *f*_1_ = *n*/60. We found that the 1-order vibration frequency equals the rotation frequency of the shaft (within 1500 rpm), and the twist vibration of the shaft may be due to the inhomogeneous shaft mass distribution or the inhomogeneous additional torques produced by the driving system and the load.

## 4. Conclusions

We have proposed an optical fiber sensing system for the torque and torsion angle of rotating shafts using two FBGs and high-speed wavelength demodulation. The dependence relationship of the dynamic difference of central wavelengths of two FBGs pasted on the surface of the circular shaft along the ±45° direction on the torque and torsion angle of the rotating shaft have been obtained. A serial of vibration experiments shows that the measured data are approximately consistent with the theoretical analysis. The difference of two central wavelengths can be divided into two parts: A “DC” part and a harmonic “AC” part. The “DC” part of the difference of two central wavelengths shows a good linear fit with the driving torque and the 1-order frequency of the harmonic “AC” part increases with the increasing rotation speed.
